# Association of serum calcium levels with renal impairment and all-cause death in Chinese patients with newly diagnosed multiple myeloma: a cross-sectional, longitudinal study

**DOI:** 10.1186/s12986-020-00525-0

**Published:** 2021-02-11

**Authors:** Jun Cheng, Wen Zhang, Yi Zhao, Xiayu Li, Rong Lv, Heng Li, Jianghua Chen

**Affiliations:** 1 Kidney Disease Center, The First Affiliated Hospital, Medical School of Zhejiang University, Hangzhou, 310003 China; 2 Department of Nephrology, The Yuhang District First People’s Hospital, Hangzhou, China; 3 Hematology Center, The First Affiliated Hospital, Medical School of Zhejiang University, Hangzhou, 310003 China

**Keywords:** Multiple myeloma, Serum calcium level, Renal impairment, End-stage renal disease, Mortality

## Abstract

**Background:**

More studies have shown that serum calcium has a crucial role in many types of cancers. However, few studies have determined the association between serum calcium levels and renal impairment (RI) and all-cause death in Chinese patients with multiple myeloma (MM).

**Methods:**

A total of 246 of 565 participants who were followed for > 6 months from a MM cohort at our institution were eligible for the retrospective study. A generalized additive model and smooth curve fitting were performed to investigate the cross-sectional relationship between the serum calcium level and RI at baseline. Multivariate-adjusted Cox regression models were fitted to assess the associations between baseline serum calcium levels and the onset of end-stage renal disease (ESRD) or death in patients with MM.

**Results:**

A total of 172 of 565 patients (30.4%) with newly diagnosed MM presented with RI. The mean duration of follow-up was 26.64 months. Twenty-one patients (8.54%) died and 28 patients (11.52%) had ESRD. In patients with a serum calcium level > 2.30 mmol/L, the serum calcium level was independently associated with the occurrence of MM-related RI. Cox regression analysis showed that baseline serum calcium levels were consistently associated with a higher risk of all-cause death in the fully adjusted model, but were not associated with the occurrence of ESRD. When patients were categorized into two groups according to baseline mean serum calcium level, deaths occurred in 13 patients (15.1%) with a mean serum calcium level > 2.44 mmol/L compared to eight patients (5.0%) with a mean serum calcium level < 2.44 mmol/L (*p* < 0.05); Eighteen patients (11.46%) with a mean serum calcium level < 2.44 mmol/L progressed to ESRD compared to 13 patients (11.6%) with a serum calcium level > 2.44 mmol/L (*p* > 0.05).

**Conclusions:**

This observational study showed that there was a nonlinear relationship between the serum calcium level and the presence of RI in patients with MM. An elevated baseline calcium level predicted all-cause death, but did not predict the occurrence of ESRD in patients with MM followed for > 6 months.

## Background

Multiple myeloma (MM) is a clonal B-cell malignancy of the bone marrow that is associated with a variety of clinical manifestations, including hypercalcemia, renal impairment (RI), anemia, and bone disease. MM is the second most common hematologic malignancy and accounts for 1% of all malignancies [1].

RI is a common complication of MM. Depending on the definition of RI (defined as a serum creatinine level > 2 mg/dL), this complication is reported in 15–40% of patients with MM [2]. RI can predict a poor prognosis in patients with MM. In fact, recent studies from Ireland [3] and the UK [4] have shown that survival in dialysis-dependent patients during the first weeks after diagnosis of MM has not improved substantially in recent years, despite the availability of novel drugs.

A number of studies have reported that serum calcium has a crucial role in many types of cancers, such as breast [5], ovarian [6], and prostate cancer [7]. Serum calcium is also a novel parameter with which to assess metabolic syndrome in endometrial carcinoma [8]. Myeloma bone disease can result in excess bone resorption, which causes excessive release of calcium leading to hypercalcemia (defined as a serum calcium concentration > 11.5 mg/dL or 2.85 mmol/L). Several cytokines, such as MIP-1α, RANKL, and DKK1, have significant roles in the exaggerated osteoclastic bone resorption in patients with MM [9, 10]. Hypercalcemia is a defining characteristic of symptomatic MM and observed in 20–40% of newly diagnosed patients [11, 12]. Additional studies have shown that hypercalcemia is an important cause of renal failure in patients with MM [11, 12]. At the same time, hypercalcemia is associated with inferior survival and a two-fold increase in the risk of early mortality [13].

Early mortality is defined as death by any cause within the first 6 months following pathological diagnosis of MM [14]. Infection and renal failure are the main direct causes of early mortality, but cannot be accurately predicted based on the presenting prognostic features [15–17].

The main shortcoming with the existing research is that these studies only focused on the effects of hypercalcemia on bad prognosis, such as early mortality. Few similar studies have been conducted to investigate the association between serum calcium levels and all-cause death and RI in MM patients who have completed > 6 months of follow-up.

Therefore, we conducted a retrospective study to identify the relationship between the serum calcium level and MM-related RI and the estimated glomerular filtration rate (eGFR) at baseline by cross-section analysis. We also further determined whether elevated calcium levels predict all-cause death and renal failure in a cohort of MM patients followed for > 6 months.

## Methods

In this single center retrospective cohort study, we collected clinical and hematological data from newly diagnosed MM patients at the First Affiliated Hospital of the Medical School at Zhejiang University from January 2011 to June 2017. MM was defined according to the International Myeloma Working Group (IMWG) criteria [14]. The exclusion criteria were as follows: 1) missing clinical data and calcium and albumin values prior to chemotherapy; 2) a history of kidney disease, severe infection, liver disease, or autoimmune disease; 3) a history of other solid tumors; and 4) follow-up for < 6 months in the longitudinal study.

Our study was approved by the Ethics Committee of the First Affiliated Hospital (reference number: 20191380).

### Data collection and laboratory measurements

Baseline demographic and clinical data were retrieved electronically from the medical records of the general hospital registry and reviewed retrospectively. For MM patients with multiple admissions, only the first set of observation data was used, thus preserving the assumption of independence of observations.

The following indicators were evaluated: 1) demographic characteristics, including age, sex, and underlying disease (hypertension and diabetes); 2) laboratory data, including hemoglobin, serum calcium, serum creatinine, albumin, globulin, serum/urine light chain protein, lactate dehydrogenase (LDH), and beta-2 microglobulin; 3) anti-myeloma therapy regimen; 4) duration of follow-up; 5) serum creatinine at the last follow-up evaluation, time of death, or dialysis; 6) serum calcium and albumin levels (Cobas Integra reagents; Roche Diagnostics, Switzerland).

Albumin-adjusted serum calcium was calculated using the following formula: serum albumin-corrected calcium (mg/dL) = total calcium (mg/dL) + 0.8 × [4 - albumin (g/dL)] [18–20].

### Investigation of study outcomes

First, we conducted a cross-sectional study to determine the relationship between the serum calcium level, eGFR, and RI. The definition of RI, according to the novel IMWG criteria for symptomatic MM, was based on an elevated sCr > 2 mg/dL or reduced creatinine clearance (eGFR < 40 mL/min). Evaluation of the eGFR was assessed by the Chronic Kidney Disease Epidemiology Collaboration (CKD-EPI) [21–23].

We also determined whether elevated serum calcium levels predicted kidney disease outcomes and all-cause deaths. If patients were not lost to follow-up or died > 6 months beyond the designated follow-up period, the information up to the final recorded visit was used.

Primary outcome was the onset of end-stage renal disease (defined as the initiation of renal replacement therapy or an eGFR < 15 ml/min) or death.

### Statistical analysis

Continuous data are expressed as the mean ± standard deviation or median. Categorical variables are presented as a number or percentage. The difference between two groups was assessed using a Student’s *t*-test, chi-squared test, or Mann-Whitney *U* test, as appropriate.

We then used a multivariable linear regression model to estimate the independent relationship between the serum calcium level and the presence of RI at baseline with an adjustment for potential confounders. A generalized additive model and smooth curve fitting (penalized spline method) were conducted to investigate the cross-sectional relationship between serum calcium level and RI and eGFR at baseline. We further used a two piecewise linear regression model to identify the nonlinear relationships. If a nonlinear correlation existed, a two piecewise linear regression model was used to calculate the threshold effect of the calcium concentration on MM-related RI in terms of the smoothing plot. When the threshold level was apparent on the smoothed curve, the inflection point was automatically calculated by the recursive method and the maximum model likelihood was used [24, 25].

A Cox proportional hazards model was used to identify independent variables for the primary end point. Results were presented as hazard ratio (HR) and 95% confidence interval (CI). In addition, time-dependent receiver operating characteristic curve (ROC) analysis was used to evaluate the prognostic value of the calcium level for the outcome. The area under the curve was calculated for the calcium level. All probabilities were two-tailed, and statistical analysis was performed using Empower Stats (www.empowerstats.com; X & Y Solution, Inc., Boston, MA, USA) and R software (http://www.R-project.org) [25]. A *p*-value < 0.05 was considered significant.

## Results

**Fig. 1:**
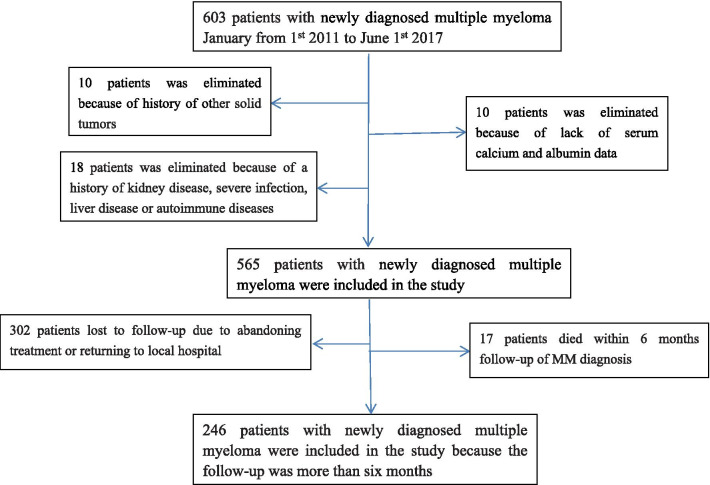
Flow chart of participants in the cohort. 565 patients were enrolled were enrolled in the cross-sectional study. Of these, 319 patients were excluded and 246 patients with newly diagnosed MM were followed for > 6 months were enrolled in the longitudinal study. Abbreviations: MM, multiple myeloma

Of the 603 participants, 38 were excluded from this study, such that 565 patients were enrolled. A total of 302 patients were lost to follow-up due to abandoning treatment or returning to a local hospital. A total of 246 patients with newly diagnosed MM were followed for > 6 months. The specific details of enrollment and exclusion are shown in Fig. 1. The baseline characteristics of the study population are listed in Table 1.

**Table 1:** Demographics of patients with MM

	MM patients without RI	MM patients with RI	*P*-value
N	393	172	
Age(year)	62.5 ± 10.2	64.4 ± 9.9	0.053
Sex			0.99
Female	153 (38.93%)	67 (38.95%)	
Male	240 (61.07%)	105 (61.05%)	
Hemoglobin(g/l)	96.62 ± 25.28	79.21 ± 20.74	0.001
Serum globulin(g/l)	53.51 ± 25.36	41.36 ± 25.41	0.001
Serum albumin(g/l)	35.1 ± 7.9	35.92 ± 7.87	0.051
Serum creatinine level(umol/L)	80.91 ± 26.21	421.62 ± 298.00	0.001
Serum calcium(mmol/l)	2.27 ± 0.25	2.46 ± 0.48	0.001
Albumin-adjusted serum calcium (mmol/l)	2.39 ± 0.28	2.57 ± 0.48	0.001
Serum kappa light chain(mg/dl)	2546.57 ± 3326.10	1586.37 ± 2428.69	0.148
Serum lambda light chain(mg/dl)	1440.24 ± 2192.25	1260.52 ± 2512.80	0.148
Urinary kappa light chain(mg/dl)	107.22 ± 285.70	357.53 ± 845.66	0.001
Urinary lambda light chain(mg/dl)	330.82 ± 1150.39	313.98 ± 691.86	0.001
Serum beta 2 microglobulin(ug/l)	4963.87 ± 4176.80	16,212.05 ± 8395.89	0.001
Lactate dehydrogenase(u/l)	192.63 ± 125.68	234.43 ± 157.08	0.001

### Relationship between the serum calcium level and the occurrence of RI based on cross-section analysis

The mean age was 63.1 ± 10.1 years; 61.1% of the 565 patients were men with newly diagnosed MM. Using the IMWG criteria, 172 of the 565 patients (30.4%) with newly diagnosed MM presented with RI.

The median serum calcium level was 2.45 mmol/L. Hypercalcemia was noted in 11.2% of newly diagnosed MM patients when the serum calcium level was albumin-adjusted, but the incidence of hypercalcemia was 7.3% of newly diagnosed MM patients if the serum calcium level was not albumin-adjusted. The incidence of hypercalcemia in this study was lower than the 20–40% reported in the literature [26, 27].

Correlation analysis revealed that the serum calcium level was positively correlated with RI (*r* = 0.22, *p* < 0.001), the serum globulin level (*r* = 0.21, *p* < 0.001), the serum lambda light chain level (*r* = 0.11, *p* = 0.016), the serum beta-2 microglobulin level (*r* = 0.23, *p* < 0.001), and ISS (international staging system) stage (*r* = 0.24, *p* < 0.001), and negatively correlated with the eGFR (*r* = − 0.27, *p* < 0.001), hemoglobin level(HB)(r = − 0.24, *p* < 0.001), and albumin level (*r* = − 0.34, *p* < 0.001).

Based on multivariable linear regression analysis adjusted for these variables, the serum calcium level was independently associated with RI (Table 2).

**Table 2:** Cross-sectional Correlation Analyses between serum calcium level and the presence of RI in different models

Variable	The occurrence of RI		
	Crude model (HR, 95%CI, P)	Minimally adjusted model(HR, 95%CI, P)	Fully adjusted model (HR, 95%CI, P)
Serum calcium level(mmol/l)	3.0 (1.9, 5.0) < 0.001	3.0 (1.8, 4.9) < 0.001	3.6 (1.8, 7.1) < 0.001
Mean serum calcium(mmol/l)			
< 2.45	1.0	1.0	1.0
> =2.45	1.7 (1.2, 2.5) 0.004	1.7 (1.2, 2.5) 0.005	1.8 (1.1, 3.1) 0.016

Nonadjusted model adjust for: None Adjust I model adjust for: age; sex.; hypertension history; diabetes history

Adjust II model adjust for: age; sex; hypertension history; diabetes history, LDH, HB, serum globulin and ISS-stage, serum albumin, serum/urinary kappa light chain, serum/urinary lambda light chain and serum beta 2 microglobulin

**Fig. 2:**
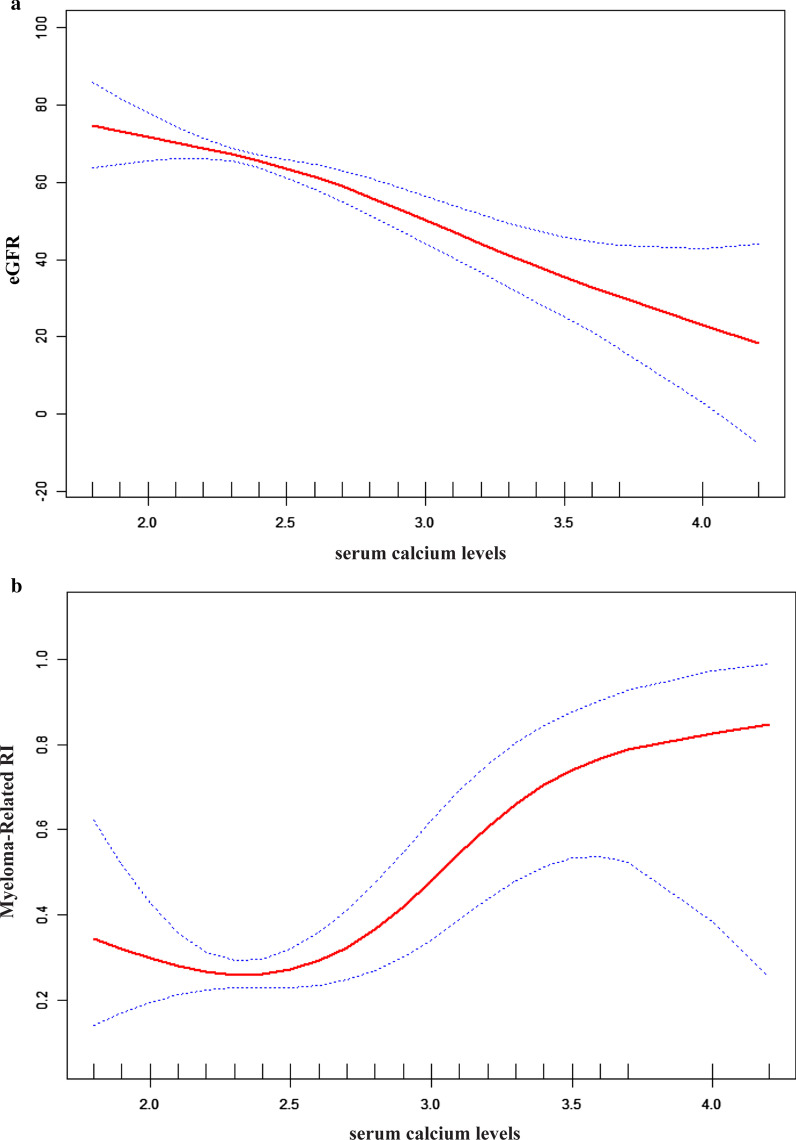
Cross-sectional associations of serum calcium level with estimated glomerular filtration rate (eGFR) (**a**) and Myeloma-Related RI (**b**) by using in a generalized additive model and smooth curve fitting. There was a negative correlation between the serum calcium level and the eGFR (*p* < 0.01). A nonlinear relationship about serum calcium level versus RI was detected (*p* < 0.01) after adjusting for age; sex, LDH, urinary kappa/Lambda light chain, serum kappa/Lambda light chain, HB, ISS-stage, serum albumin, serum beta 2 microglobulin

Figure 2 is a smoothing plot of the serum calcium level versus RI and eGFR. The curve shows that there was a negative correlation between the serum calcium level and the eGFR, and the relationship between the serum calcium level and RI was not simply linear. Specifically, as shown in Table 3, threshold effect analysis indicated that the incidence of RI increased as the serum calcium level increased, up to 2.30 mmol/L. In patients with a serum calcium level < 2.30 mmol/L, the serum calcium level was not significantly associated with RI in MM patients (*r* = 0.10, *p* = 0.108). In patients with a serum calcium level > 2.30 mmol/L, the correlation coefficient of RI was positive (*r* = 6.2, *p* < 0.001).

**Table 3:** Threshold effect analysis of serum calcium level and the presence of RI using piece-wise linear regression

Model	Result [β (95%CI) *P* value]
Model I	
one-line linear regression model	2.80 (1.4, 5.6) 0.004
Model II	
turning point	2.30 mmol/L
Group1 < 2.3 correlation coefficient (β1)	0.10 (0.0, 2.3) 0.108
Group2 > 2.3, correlation coefficient (β1)	6.20(1.9, 10.2) < 0.001
predictive value of RI at turning point	−1.40 (−1.7, −1.1)
a log likelihood ratio test	0.011

Effect: albumin-adjusted serum calcium level; Cause: myeloma-related RI adjusted for age; sex; hypertension history; diabetes history LDH, serum/urinary kappa light chain, serum/urinary lambda light chain, HB, serum globulin and ISS-stage, serum albumin, serum beta 2 microglobulin

### Serum calcium level, ESRD, and all-cause death

To evaluate the prognostic value of the serum calcium level in MM patients followed for > 180 days, patients were categorized into 2 groups according to the mean serum calcium level (2.44 mmol/L), and the clinical outcomes were compared (Table 4).

**Table 4:** Clinical Outcomes according to mean serum calcium level

Variable	mean serum calcium level < 2.44umol/L	mean serum calcium level > 2.44umol/L	*P*-value
N	160	86	
Age(year)	60.7 ± 9.8	62.7 ± 9.6	0.197
Follow-up time(month)	27.4 ± 18.8	25.2 ± 15.1	0.730
Sex			0.23
female	62 (38.8%)	31 (36.0%)	
Male	98 (61.3%)	55 (64.0%)	
ESRD.			0.970
No	139 (88.5%)	76 (88.4%)	
Yes	18 (11.5%)	10 (11.6%)	
All-cause death.			0.015
No	152 (95.0%)	73 (84.9%)	
Yes	8 (5.0%)	13 (15.1%)	

Patients who were followed for at least 6 months were included in the study. A total of 246 patients were studied in this observational cohort. The mean age was 61.41 ± 9.73 years and 62.20% were men. The mean duration of follow-up was 26.64 months. Twenty-one patients (8.54%) died and 28 patients (11.52%) had ESRD 6 months after diagnosis. Of these patients, 18 (11.46%) with a mean serum calcium level < 2.44 mmol/L developed ESRD compared with 13 patients (11.6%) who had a serum calcium level > 2.44 mmol/L (*p* > 0.05). Moreover, all-cause deaths occurred in 13 patients (15.1%) with a mean serum calcium level > 2.44 mmol/L and 8 patients (5.00%) with a mean serum calcium level < 2.44 mmol/L (*p* < 0.05; Table 4).

Correlation analyses based on univariate regression revealed that all-cause deaths correlated with the serum calcium level (*r* = 0.1875, *p* < 0.001), Lactate dehydrogenase (LDH)(*r* = 0.1604, *p* < 0.001), RI (*r* = 0.1382, *p* = 0.05) and eGFR (*r* = − 0.1608, *p* = 0.01).

**Fig. 3:**
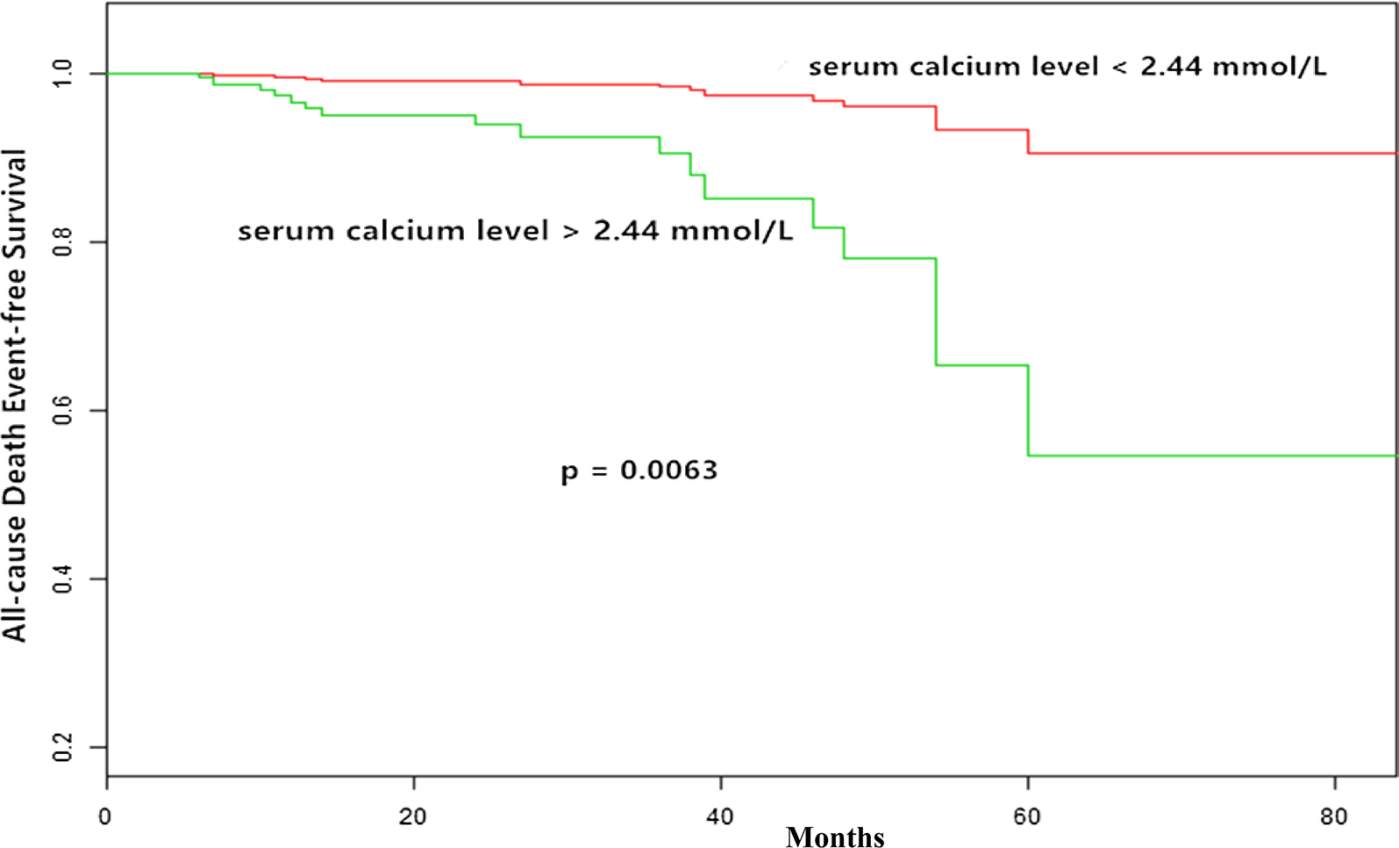
Kaplan-Meier curves of all-cause death outcome according to mean serum calcium levels .Compared with patients with serum calcium levels> 2.44 mmol/L, all-cause death was significantly lower in patients with serum calcium levels< 2.44 mmol/L (*P =* 0.0063)

In Cox regression analysis adjusted for demographic and clinical factors of age, sex, an increase in the serum calcium level was significantly associated with an increase in the risk of the all-cause death (HR, 4.82; 95% CI, 2.37–9.78; *p* < 0.001; Table 5 [model 1]). Furthermore, the fully adjusted model, including RI, LDH, HB, albumin, serum beta 2 microglobulin, serum /urinary kappa or lambda light chains, and anti-myeloma therapy regimen, showed a significant increase in risk of adverse outcomes conferred by the serum calcium level (HR, 5.72; 95% CI, 2.09–15.63; *p* < 0.001; Table 5 [model 2]). We also did a separate analysis in which the serum calcium level was treated as a categorical variable by the mean. The risk of all-cause death was significantly higher in patients with a mean serum calcium level > 2.44 mmol/L than in patients with a serum calcium level < 2.44 mmol/L (HR, 6.99; 95% CI, 1.61–30.41; *p* < 0.01; Table 5). A Kaplan-Meier curve revealed that event-free survival for the all-cause death outcome was significantly lower in patients with a serum calcium level > 2.44 mmol/L compared to patients with a serum calcium level < 2.44 mmol/L (*p* = 0.0063; Fig. 3).

**Table 5:** Relationship between serum calcium level and All-cause death and ESRD in different models

Variable	All-cause death			ESRD		
	Crude model (HR, 95%CI, P)	Minimally adjusted model(HR, 95%CI, P)	Fully adjusted model(HR,95%CI,P)	Crude model (HR, 95%CI, P)	Minimally adjusted model(HR, 95%CI, P)	Fully adjusted model (HR, 95%CI, P)
Serum calcium level(mmol/l)	4.82 (2.37, 9.78) < 0.0001	4.95 (2.36, 10.37) < 0.0001	5.72 (2.09, 15.63) 0.0007	1.34 (0.53, 3.37) 0.5331	1.32 (0.51, 3.40) 0.5624	0.85 (0.29, 2.48) 0.7655
Mean serum calcium(mmol/l)						
< 2.44	0	0	0	1.0	1.0	1.0
> =2.44	4.13 (1.62, 10.51) 0.0029	4.27 (1.66, 11.01) 0.0026	6.99 (1.61, 30.41) 0.0095	1.09 (0.50, 2.39) 0.8211	1.03 (0.47, 2.25) 0.9444	0.95 (0.39, 2.34) 0.9139

Non-adjusted model adjust for: None Adjust I model adjust for: age sex.; hypertension history; diabetes history

Adjust II model adjust for: age; sex.; hypertension history; diabetes history, LDH, serum/urinary kappa light chain, serum/urinary lambda light chain, HB, serum globulin and ISS-stage, serum albumin and serum beta 2 microglobulin

**Fig. 4:**
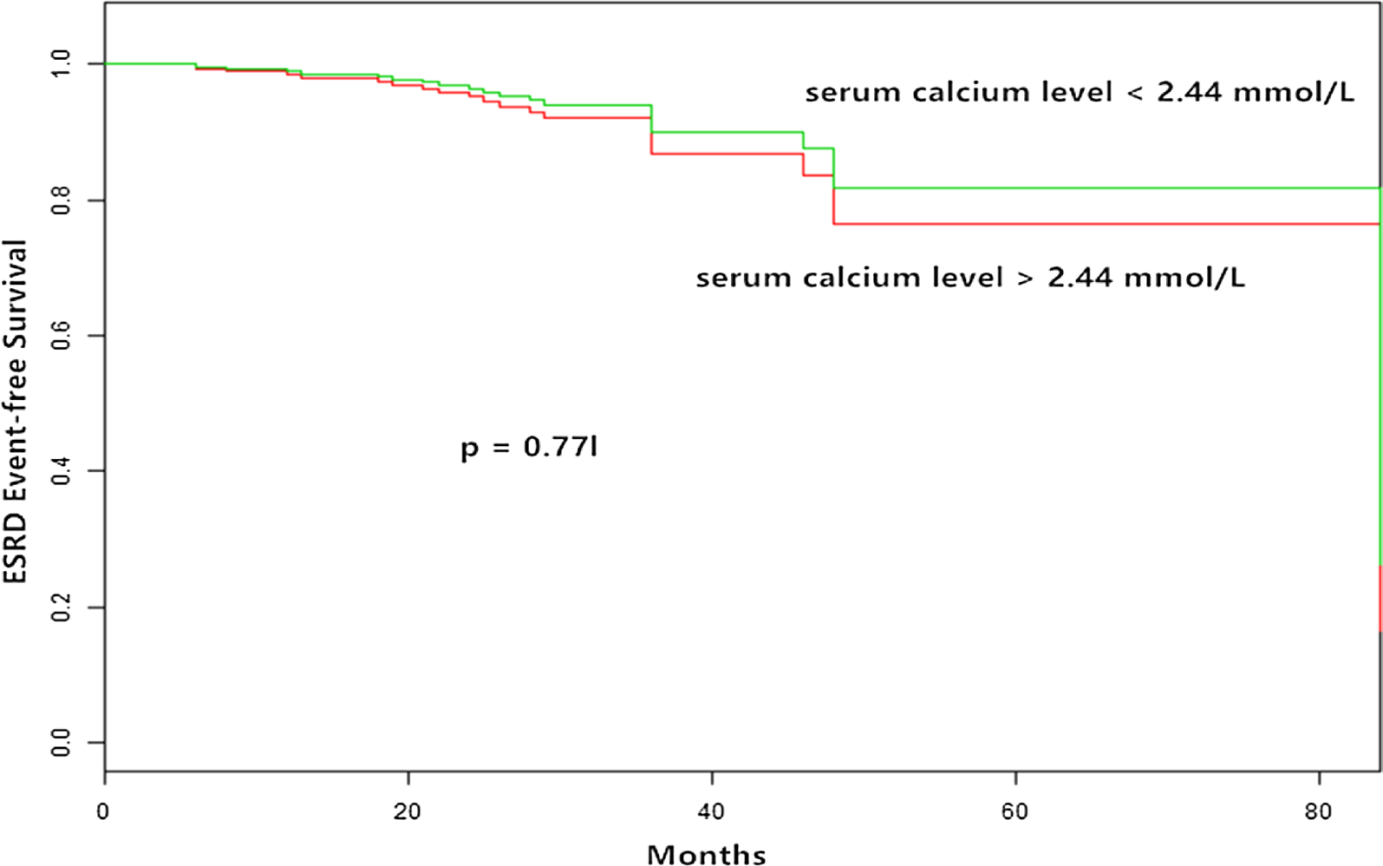
Kaplan-Meier curves of ESRD outcome according to mean serum calcium levels. Compared with patients with serum calcium levels> 2.44 mmol/L, ESRD events was no difference in patients with serum calcium levels< 2.44 mmol/L (*P =* 0.771)

In this study we showed that the serum calcium level did not predict ESRD in a Cox regression model (HR = 0.85; 95% CI, 0.29, 2.48; *p* = 0.77 l; Table 5). An increase in the serum calcium level was not associated with an increased risk of ESRD (Fig. 4).

**Fig. 5:**
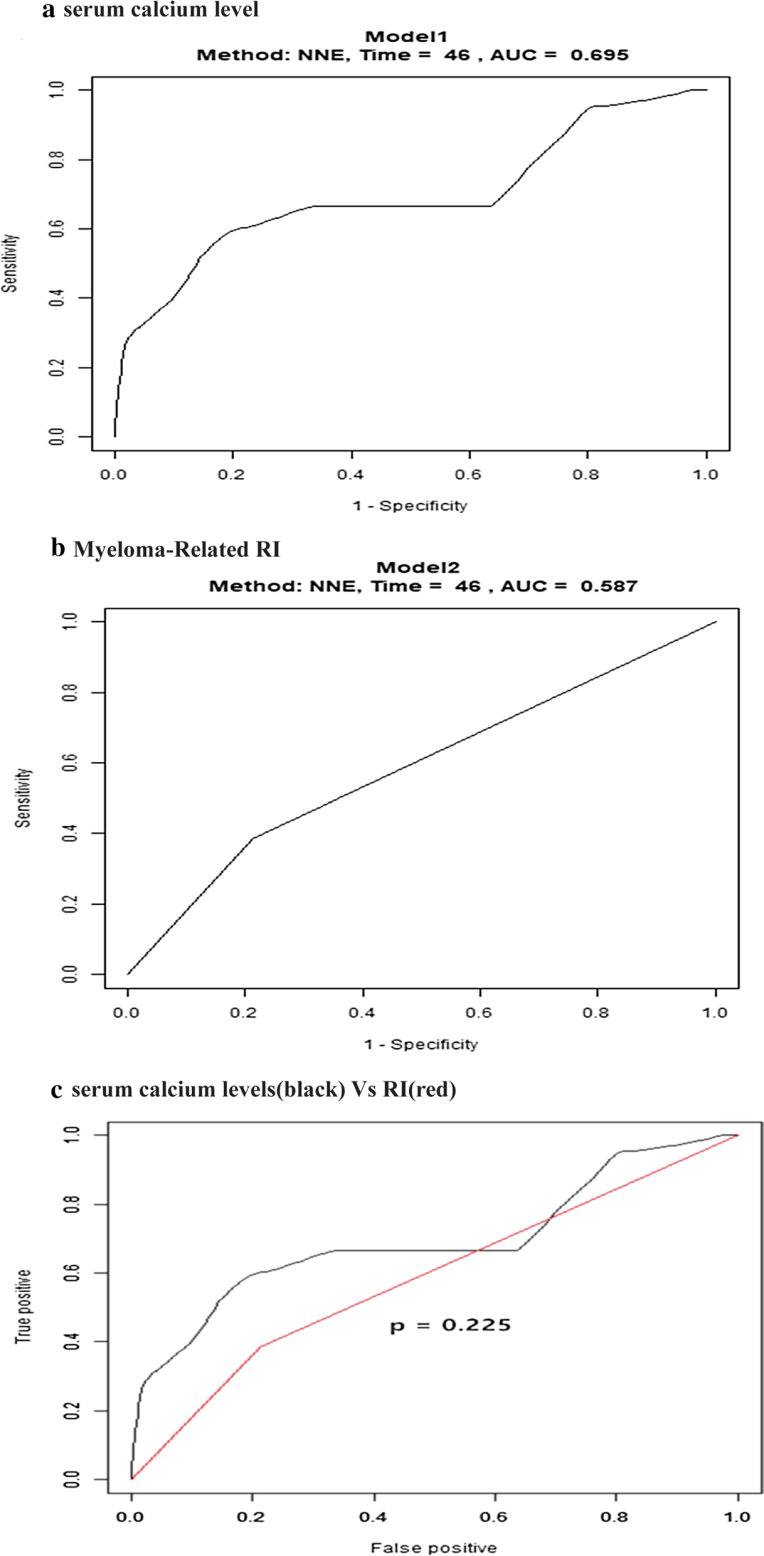
Time-dependent receiver operating characteristics curves of (**a**) serum calcium levels (**b**) Myeloma-Related RI, (**c**) serum calcium levels(black) Vs Myeloma-Related RI(red). The areas under the ROC curve for the serum calcium level were 0.695, however, the areas under the ROC curve for RI were 0.587, respectively .There was no different (*p* = 0.225)

To evaluate the operating characteristics of the serum calcium level as a prognostic value for all-cause death in MM patients followed for > 6 months, we conducted a time-dependent ROC analysis for the serum calcium level in comparison with RI (Fig. 5). The areas under the ROC curve for the serum calcium level at 12, 27, and 46 months were 0.705, 0.699, and 0.763, respectively; however, the areas under the ROC curve for RI were 0.736, 0.63, and 0.573, respectively (*p* = 0.225).

## Discussion

In this study we showed that the serum calcium level was higher in patients with MM-related RI and was associated with MM-related RI and the baseline eGFR in patients with MM. We also showed a nonlinear relationship between the serum calcium level and the presence of RI in patients with MM. We demonstrated that high serum calcium levels significantly predicted all-cause death for patients followed for > 6 months, suggesting the serum calcium level was a biomarker for the MM survival rate. We also found, however, that high serum calcium levels did not predict the occurrence of ESRD in MM patients.

RI is a common feature of MM and may provide a clue to diagnosis and cause a major management problem. This complication occurs in 20–40% of newly diagnosed patients with MM. Using the IMWG criteria,172 of 565 patients (30.4%) with newly diagnosed MM presented with RI. The 2-year all-cause mortality of patients with ESRD due to MM-related RI was 58% versus 31% in all other patients without RI [22].

Hypercalcemia is as the second most common cause of renal failure in patients with MM, following free light chains [12, 13]. Some researchers are of the opinion that hypercalcemia and/or Bence-Jones proteinuria explain renal failure in 97% of patients [12, 13]. Hypercalcemia interferes with renal function and impairs the renal concentrating ability, causes vasoconstriction of the renal vasculature, and enhances diuresis, which may result in hypovolemia and pre-renal azotemia. Concentrated urine and reduced urine flow enhance cast formation, thus leading to further renal damage.

Hypercalcemia is observed approximately in 15% of newly diagnosed patients [26, 27]. The results from 565 Chinese MM patients in this study showed that the incidence of hypercalcemia was 11.31% (albumin-adjusted) or 7.42% (albumin with adjustment). A number of studies have focused on the effects of hypercalcemia on MM-related RI and clinical outcomes [12, 13], but few studies have investigated the association between the serum calcium level and MM-related RI in patients with MM. More importantly, the clinical implication of the serum calcium level associated with ESRD and all-cause death is unknown. This is the first study to show that the serum calcium level may act as a useful prognostic marker in MM patients followed up for > 6 months. Our study clearly showed that there was a negative correlation between the serum total calcium level and the eGFR. Our finding of higher serum calcium levels in patients with a reduced eGFR is consistent with results from a recent study by Zagouri et al. [13], who reported that hypercalcemia is associated with a lower eGFR and is an independent risk factor. Our study confirmed that the serum calcium level is associated with MM-related RI. More importantly, we showed that there is a nonlinear relationship between the serum calcium level and RI. There was a positive correlation between the serum calcium level and RI when the serum calcium level was > 2.3 mmol/L (*p* < 0.05) based on our cross-sectional analysis.

ESRD, especially in patients undergoing long-term dialysis, has a worse prognosis. Unfortunately, the serum calcium level was shown to be correlated with MM-related RI, but was not an independent risk factor for ESRD in MM patients followed up for > 6 months.

Early mortality was defined as death by any cause within the first 6 months following pathologic diagnosis of MM. Early mortality after diagnosis of MM is often attributed to combined effects of active disease and co-morbid factors. Recent population studies have shown that nearly one in four patients with MM die within one year of diagnosis, and nearly half of the deaths occur within the first three months [15–17].

Infection and renal failure are the main direct causes of early mortality, which cannot be accurately predicted by presenting prognostic features [15–17]. Therefore, patients with relatively stable conditions were selected for this study, and these patients included in the study completed at least six cycles of chemotherapy.

In fact, we demonstrated that more patients with a serum calcium level greater than the mean value resulted in death compared with patients with a serum calcium level lower than the mean value. Furthermore, a higher serum calcium level was consistently associated with a higher risk of all-cause death in multivariable models after adjustment for various clinical and laboratory factors. After at least 6 months of follow-up, the mortality rate of patients with a serum calcium level greater than the mean value (2.44 μmol/L [not hypercalcemia]) was six times higher than patients with a serum calcium level below the mean value.

In the era of conventional chemotherapy (CC), RI is associated with a poor median survival time of approximately two years [28–30]. Severe RI is also associated with as increased risk of early death [31, 32]. The use of novel anti-myeloma drugs results in an increase in the survival of patients with MM and RI. We found that the areas under the ROC curves of serum calcium levels and RI did not differ. These findings implied that both the serum calcium level and RI independently predicted adverse outcomes, and the prognostic utility of these markers was similar (*p* = 0.225).

Our study had several limitations. First, the limitations of our study included the observational design, retrospective ascertainment of the serum creatinine level, the serum calcium level at a single time point late during the course of newly diagnosed MM, and the potential misclassification of study measurements. Second, after a clear diagnosis of MM, because some patients returned to the local hospital for treatment, some patients were followed for < 6 months, so they were not included in this study. Third, whether the albumin correction is required for total calcium in peripheral blood has been controversial in recent years. This study was mainly based on albumin correction; however, we also analyzed total calcium without albumin correction, and the results did not change.

## Conclusions

In summary, our study showed that the baseline serum calcium level was associated with RI in Chinese patients with newly diagnosed MM. There was a nonlinear relationship between the serum calcium level and RI. An elevated baseline calcium level predicted all-cause death in patients with MM, but did not predict the occurrence of ESRD, suggesting that an elevated serum calcium level may serve as a useful clinical biomarker for the survival MM patients followed for > 6 months. However, our findings were hypothesis-generating based on results of retrospective observational studies; thus, further studies with a larger number of patients are required to validate our findings.

## Data Availability

The datasets used and/or analysed during the current study are available from the corresponding author on reasonable request.

## Abbreviations

MM: Multiple myeloma
RI: Renal impairment
IMWG: International Myeloma Working Group
ISS stage: International staging system
LDH: Lactate dehydrogenase
GFR: Glomerular filtration rate
CIs: Confidence intervals
